# Population viability in a host-parasitoid system is mediated by interactions between population stage structure and life stage differential susceptibility to toxicants

**DOI:** 10.1038/s41598-020-77496-y

**Published:** 2020-11-27

**Authors:** John D. Stark, Jenifer K. McIntyre, John E. Banks

**Affiliations:** 1 Ecotoxicology Program, Department of Entomology, Research and Extension Center, Washington State University, Puyallup, WA 98371 USA; 2 Research and Extension Center, School of the Environment, Washington State University, Puyallup, WA 98371 USA; 3 Undergraduate Research Opportunities Center, California State University, 100 Campus Center Seaside, Monterey Bay, CA 93955 USA

**Keywords:** Environmental impact, Ecology

## Abstract

The effects of toxicants, such as pesticides, may be more severe for some life stages of an organism than others. However, in most toxicity studies, data is developed for only one life stage, which may lead to misleading interpretations. Furthermore, population stage-structure may interact with differential susceptibility, especially when populations consist of higher proportions of individuals in more susceptible stages at the time of toxicant exposure. We explore the interaction of differential stage susceptibility and stage distribution using a stage-structured Lefkovitch matrix model. We incorporate lab-derived toxicity data for a common parasitoid, the braconid *Diaeretiella rapae* (M’Intosh), a common natural enemy of the cabbage aphid (*Brevicoryne brassicae* L.), exposed to the pesticide imidacloprid. We compare population outcomes of simulations in which we vary both the population stage structure along with the susceptibility of each stage to toxicants. Our results illustrate an interaction between differential susceptibility and initial stage distribution, highlighting the fact that both of these demographic features should be considered in interpreting toxicity data and the development of ecological risk assessments.

## Introduction

The compatibility of pesticides and other toxicants with natural enemies is critical to the success of biological control and integrated pest management schemes^1–3^. Because predator and prey populations often respond differently to toxicants, pesticide exposure may disrupt predator–prey interactions and facilitate pest population outbreaks^4–7^. Different natural enemy species may exhibit widely variable population responses to toxicants; variations in life history strategies and population structure may be important drivers of natural enemy responses to pesticides^8^.

Lab and field-based studies have shown that some life stages within a species are more susceptible to pesticides and other toxicants than others^1,9–19^. However, in most toxicity studies, toxicity data are developed for only one life stage. An important question is thus: if we have toxicity data for only one life stage, and differential stage susceptibility occurs, how accurately can these data predict overall effects on populations? Furthermore, an important consideration with regard to differential stage susceptibility is the structure of a population at the time of exposure to a toxicant, such as in a pesticide application. In particular, populations with larger vs. smaller proportions of susceptible stage individuals in a population might yield very different population outcomes when exposed to toxicants. Despite advances in our understanding of how demography affects population outcomes in response to toxicants, differential susceptibility and population structure is rarely (if ever) considered when risk assessments for chemicals are developed. These factors may be particularly important for pest managers when they are trying to determine whether a particular pesticide is compatible with a biological control organism.

We present here a modeling approach to testing the effects of population responses to toxicants in which we vary (1) differential susceptibility among life stages, and (2) the starting population stage structure in combination with differential stage susceptibility. Toxicity data were developed in the laboratory for two life stages, mummy and adult, of *Diaeretiella rapae* (M’Intosh), a common parasitoid of the cabbage aphid, *Brevicoryne brassicae* (L.), after exposure to the insecticide imidacloprid.

Population models based on differential stage susceptibility were developed for four starting population structures: (1) stable age, (2) a population weighted towards the adult stage, (3) a population weighted towards the young stages, and (4) a population with an equal stage distribution. Outcomes for each of these scenarios were then compared in an attempt to determine the overall effects of initial stage structure and differential susceptibility on long-term population responses to toxicant exposure.

## Results

**Figure 1:**
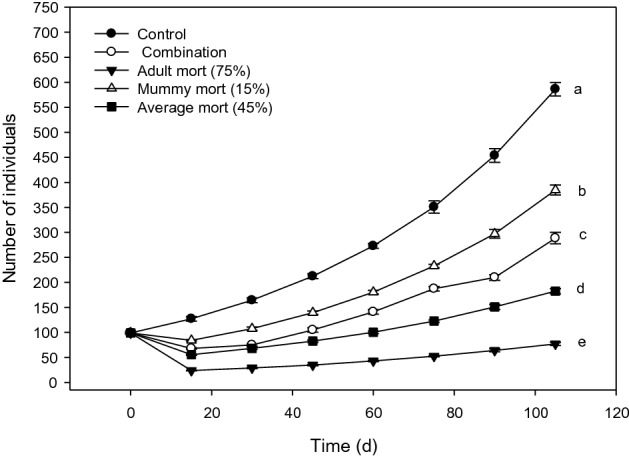
Population trajectories for *D. rapae* for simulations with initial stable stage distribution for four mortality scenarios and a control. Final population sizes labeled by different letters were statistically different. Figure created with Sigma Plot software, version 14.0.

**Figure 2:**
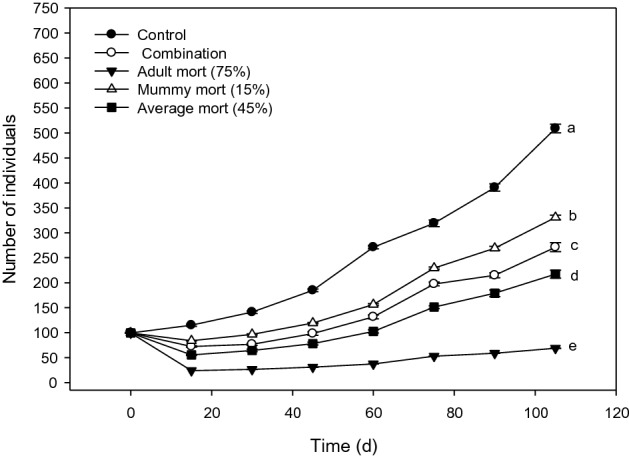
Modeled population trajectories for *D. rapae* population that started in the young-weighted stage distribution after exposure to various mortality scenarios. Final population sizes labeled by different letters were statistically different. Figure created with Sigma Plot software, version 14.0.

**Figure 3:**
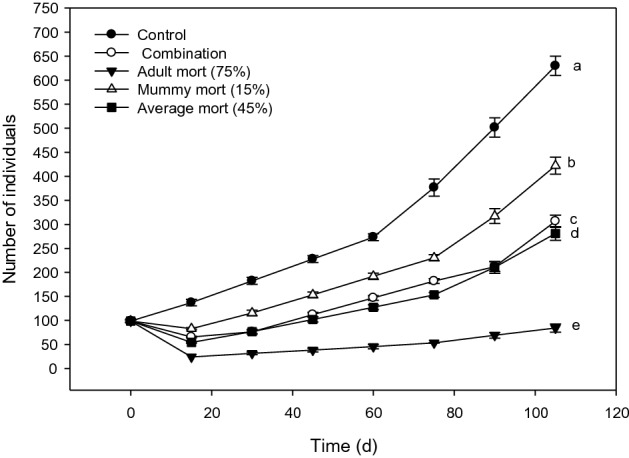
Modeled population trajectories for *D. rapae* population that started in the equally-weighted stage distribution after exposure to various mortality scenarios. Final population sizes labeled by different letters were statistically different. Figure created with Sigma Plot software, version 14.0.

**Figure 4:**
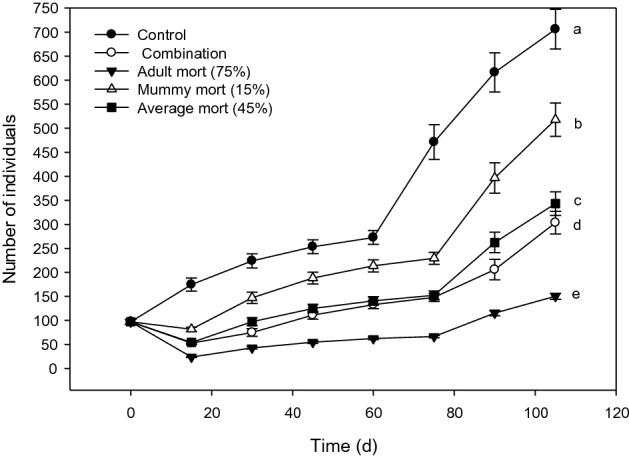
Modeled population trajectories for *D. rapae* population that started in the adult-weighted stage distribution after exposure to various mortality scenarios. Final population sizes labeled by different letters were statistically different. Figure created with Sigma Plot software, version 14.0.

Population trajectories differed depending on the mortality value assigned to each of the stage-weighted populations. In particular, for stable, young-, and equally-weighted stage distributions, the order of the most negatively affected population to least affected population was adult mortality > average mortality > combination mortality > mummy mortality (Figs. 1, 2, 3). For the adult-weighted stage distribution, the pattern was different, with adult mortality > combination mortality > average mortality > mummy mortality (Fig. 4). These results highlight the importance of considering the initial population stage structure in inferring how exposure to toxicants may affect population outcomes.

A comparison of the four starting stage distributions as a percent of their respective controls indicated that stage distribution had a significant effect on population susceptibility. The order of susceptibility from most negatively affected population to least affected after exposure to combination mortality was adult-weighted > stable-weighted = equally weighted > young weighted. However, exposure to mummy mortality resulted in the following pattern: stable-weighted = young-weighted > equally weighed > adult weighted (Table 1). Exposure to the average and adult mortalities resulted in the same pattern: stable-weighted > young-weighted > equally weighed > adult-weighted (Table 1). These results indicate that the influence of starting population structure on population susceptibility when differential stage susceptibility occurs is not intuitive.

**Table 1:** Comparison of final number of individuals as % of controls among various stage distributions and mortality levels.

Stage distributions	Combination^a^ mortality (15% mummy & 75% adult)	Mummy mortality (15%)	Average mortality (45%)	Adult mortality (75%)
Stable stage	49.2 ± 1.28b	65.6 ± 0.58c	31.1 ± 0.42d	13.1 ± 0.26d
Young weighted	53.3 ± 1.57a	65.0 ± 0.69c	42.7 ± 0.87c	18.6 ± 0.35c
Adult weighted	43.1 ± 3.74c	73.3 ± 0.89a	48.6 ± 0.79a	21.3 ± 0.51a
Equally weighted	48.7 ± 1.87b	67.0 ± 0.78b	44.6 ± 0.90b	19.4 ± 0.18b

^a^Means within a column followed by different letters are significantly different (*p* < 0.05; Tukey test).

## Discussion

We developed population models in this study for a parasitoid species, *D. rapae*, that exhibits differential stage susceptibility to the insecticide, imidacloprid. We tested the effects of population responses to imidacloprid by varying differential susceptibility among two life stages, mummy and adult, and the starting population stage structure in combination with differential stage susceptibility. In the combination mortality scenario, we incorporated toxicity data derived for the mummy and adult stage, which we considered to be the most accurate in terms of effects on a *D. rapae* population. The combination mortality scenario was considered the most accurate because it contained toxicity data that was actually developed for the two life stages, whereas in the other scenarios, toxicity data developed for only one life stage or an average of the data for the two life stages was evaluated. However, our results show that if we were working with toxicity data for only the mummy stage, then we would underestimate the population effects of imidacloprid, i.e., the model predicts the final population would be significantly higher using mummy-only mortality data than using the more realistic outcome of mummy plus adult stage mortality (Table 1). Likewise, using only adult mortality data would yield an overestimate, with population effects of imidacloprid exposure much more severe than those resulting from adult plus mummy stage data. Even the average mortality value, which reflected the mean of the mummy and adult mortality, tended to overestimate the effects of imidacloprid on *D. rapae*. Taken together, our results make a clear case for considering population structure and life stage susceptibility explicitly in risk assessment.

Differential susceptibility among species to pesticides and other toxicants is well-documented^20,21^. Likewise, within species or even with a single organism, different life stages may exhibit differential susceptibility to some toxicants (but not all); this has been exploited in order to optimize timing of pesticide exposure in biological control^9,22–24^. Our results in the current paper suggest a similar need for life stage structure and composition when considering predator effectiveness; in particular, we demonstrate that when differential susceptibility occurs for natural enemies, the population structure at the time of toxicant exposure is a critical consideration in predicting population outcomes. Although traditional static metrics such as the LC_50_ can detect differential susceptibility among life-stages (e.g.,^24^), our results emphasize the need to move beyond simply generating individual-level endpoints in assessing the impact of toxicants on exposed populations. In particular, by manipulating the composition of life stages as well as the initial conditions in our simulations, we were able to detect population consequences of differential life-stage susceptibility. These results contribute to the growing body of evidence suggesting that a population-level perspective is critical for understanding the nuances of how economically important species react to toxicant exposure^4,25–30^. We suggest that this perspective, coupled with more detailed population-structured models of differential susceptibility that incorporate pesticide/toxicant resistance (e.g.,^31,32^) points the way forward in developing optimal resource management schemes and the development of ecological risk assessments.

## Materials and methods

### Toxicity of imidacloprid to *D. rapae* life stages

#### Toxicity of imidacloprid to immature *D. rapae* inside mummified B. brassicae

Exposure of *D. rapae* immature stages and adults followed the protocol outlined by Acheampong and Stark^33^. Individual broccoli leaves containing batches of 50 mummified (pupal stage) *B. Brassicae*, parasitized by *D. rapae*, were placed in a petri dish over a moist section of paper towel. Several concentrations of imidacloprid (Admire Flowable Systemic Insecticide, Bayer Crop Science Inc. 240 g active ingredient (ai)/L) were applied with a Potter tower. The Admire field rate (FR) for control of aphids was 50 g ai imidacloprid/ha. The following concentrations were applied: 1/8 FR (6.25 g ai/ha), ¼ FR (12.5 g ai/ha), and 1/2 FR (25 g ai/ha) and FR, 50 g ai imidacloprid/ha in addition to a control (water). Each leaf received a treatment of 300 µl. Emergence of *D. rapae* was recorded for two weeks. This experiment was replicated five times.

#### Toxicity of imidacloprid to adult *D. rapae*

Adult *D. rapae* were exposed to imidacloprid-treated broccoli leaves which had been sprayed as described above in the section on mummified *B. brassicae*. Batches of 10 adult *D. rapae* approximately 24 h old were chilled in a refrigerator for 3 min and transferred to broccoli leaves. The leaves and *D. rapae* adults were placed in a Potter chamber and imidacloprid was applied as described above and at the same rates. Adult mortality was evaluated 24 h after imidacloprid exposure. This experiment was replicated five times.

### Simulations based on ¼ field rate exposure

Stochastic simulation models based on a stage-structured Lefkovitch projection matrix^34–36^ were developed to estimate the impact that exposure to imidacloprid would have on population outcomes of *D*. *rapae*. The model consisted of a primary matrix that contained the life-history characteristics (survivorship and fecundity) of a *D. rapae* population, which we published previously^37,38^ (Table 2). A starting vector, *n*(*t*), containing information on the stage distribution of the population to be evaluated, was multiplied with the primary matrix, resulting in a secondary vector, *n*(*t* + 1), that was then multiplied with the matrix and so on, with each multiplication projecting the population growth for the next time step of the matrix.

**Table 2:** *D. rapae* vital rates used in the matrix models (from^38^).

Stage	Survival rate (*Sx*)	Fecundity (*Fx*)
Egg	1.000	0
Larval	1.000	0
Pupal	1.000	0
Adult	0.100	2.545

We assumed that a host (aphid) population was established in the field prior to arrival of *D. rapae*. When imidacloprid was applied at the ¼ field rate, we further assumed that correspondingly ¼ of the aphids in the egg and larval stages died, and thus that proportion of the population could not serve as a host for *D. rapae*. These reductions were incorporated in the initial vectors.

For each simulation we set the starting population size of *D. rapae* at 100 individuals, and we calibrated the time step used in the model to the generation time of *D. rapae* under the conditions of this study (15 d). Each simulation was run for seven-time steps (105 d) to represent a summer growing season in the northern hemisphere. The effects of imidacloprid on life stages were incorporated in the starting vector, not the vital rates in the matrix, in order to simulate an acute effect on populations. That is, *D. rapae* vital rates were assumed to be unaffected by the pesticide application. Each model was designed so that changes in the levels of mortality by life stage could be manually assigned to the starting vector to evaluate the effect of differential susceptibility among life stages. Only the effects caused by the 1/4 field rate application of imidacloprid were considered in the models (Tables 3, 4). Therefore, mortality of adults emerging from the mummy stage (15%) and adult mortality (75%) were incorporated into the models. An average mortality (45%) was tested as well.

Four sets of simulations were developed based on the effects of a ¼ field rate exposure of imidacloprid to *D. rapae* (Tables 3, 4). These simulations test the effect of assuming that the mortality determined for one life stage applies to the entire population. In the first set of simulations, (“mummy”), the effect of imidacloprid found empirically in the mummy stage (15% mortality) was imposed on all life stages (Table 3). In the second set of simulations (“adult mortality”), the effect of imidacloprid on the adult stage (75% mortality) was imposed on all life stages (Table 4). In the third set of simulations (“average mortality”), the average of the toxicity of imidacloprid to the mummy and adult stage (45%) was imposed on all life stages. Finally, a fourth set of simulations (“combination”) incorporated mummy mortality (15%) in the mummy stage and adult mortality (75%) in the adult stage. In all of these models the adult stage along with the other life stages (egg and larval) were reduced 25% to account for a 25% reduction in the number of the aphid hosts of *D. rapae* due to exposure to the ¼ field rate of imidacloprid.

**Table 3:** Emergence of *D. rapae* from *B. brassicae* mummies after exposure to imidacloprid.

Treatment	% adult emergence X + SD
Control	94 ± 10
1/8 Field rate (6.25 g ai/ha)	94 ± 9
1/4 Field rate (12.50 g ai/ha)	85 ± 10
1/2 Field rate (25 g ai/ha)	68 ± 18
Field rate (50 g ai/ha)	50 ± 20

**Table 4:** Mortality of adult *D. rapae* exposed to imidacloprid.

Treatment	Mean % adult mortality + SD
Control	0 ± 0
1/8 Field rate (6.25 g ai/ha)	67 ± 8
1/4 Field rate (12.50 g ai/ha)	75 ± 8
1/2 Field rate (25 g ai/ha)	98 ± 2
Field rate (50 g ai/ha)	100 ± 0

### Effects of different starting population structure on population growth when differential stage susceptibility occurs

Four initial stage distributions of the starting vector were used for the development of models for control populations and *D. rapae* populations exposed to the ¼ field rate of imidacloprid (Table 5). The stage distributions were: (1) Stable stage distribution as determined from the control matrix model, hence called the “stable” distribution (2) a population distribution weighted towards adults, hence called the “adult” distribution, (3) a population distribution weighted towards the egg stage, hence called “young” distribution, and (4) a population distribution that had equal distributions in each stage, hence called the “equal” distribution (Table 5). Mortality, once again, was incorporated in the starting vectors only.

**Table 5:** Stage distributions of the control populations used in the initial vector in population models.

Stage	Stable stage distribution (%) (Stable distribution)	Weighted towards adults (%) (Adult distribution)	Weighted towards egg stage (%) (Young distribution)	Weighted equally (%) (Equal distribution)
Egg	34	10	50	25
Larval	27	15	25	25
Pupal	21	25	15	25
Adult	18	50	10	25
Total individuals	100	100	100	100

Separate simulations were developed for each replicate from the exposure data. A total of five simulations for each of the four initial stage distributions or 20 separate simulations for exposure to ¼ field rate of imidacloprid were run each for effects on adults only, effects on mummies only, and effects on both stages, for a total of 60 simulations. Twenty separate simulations were developed for the control populations (four starting distributions × five replicates). Final population sizes from each replicate were averaged across simulations and then statistically analyzed for differences.

Models were developed using RAMAS Metapop 6 software. Each model was parameterized as follows: models were stochastic, starting population size was 100 individuals, each model was run 1000 times with Monte Carlo resampling. Because the differently structured populations were started with different original numbers after the hypothetical exposure to imidacloprid, the final population size in each distribution was compared to their respective control (% control).

### Statistical analysis

The final population size (number of individuals) after 105 d within a stage distribution and the final population size among the different starting stage distributions as a percentage of their respective controls were compared with analysis of variance^39^. Means were separated with Tukey’s test (*p* < 0.05).

## Data Availability

All data developed for and used in this study is available upon request of the authors.
